# Phosphatidylserine is a marker for axonal debris engulfment but its exposure can be decoupled from degeneration

**DOI:** 10.1038/s41419-018-1155-z

**Published:** 2018-11-02

**Authors:** Vered Shacham-Silverberg, Hadas Sar Shalom, Ron Goldner, Yarden Golan-Vaishenker, Neta Gurwicz, Irena Gokhman, Avraham Yaron

**Affiliations:** Department of Biomolecular Sciences, Weizmann Institute of Science, Rehovot, 76100 Israel

## Abstract

Apoptotic cells expose Phosphatidylserine (PS), that serves as an “eat me” signal for engulfing cells. Previous studies have shown that PS also marks degenerating axonsduring developmental pruning or in response to insults (Wallerian degeneration), but the pathways that control PS exposure on degenerating axons are largely unknown. Here, we used a series of in vitro assays to systematically explore the regulation of PS exposure during axonal degeneration. Our results show that PS exposure is regulated by the upstream activators of axonal pruning and Wallerian degeneration. However, our investigation of signaling further downstream revealed divergence between axon degeneration and PS exposure. Importantly, elevation of the axonal energetic status hindered PS exposure, while inhibition of mitochondrial activity caused PS exposure, without degeneration. Overall, our results suggest that the levels of PS on the outer axonal membrane can be dissociated from the degeneration process and that the axonal energetic status plays a key role in the regulation of PS exposure.

## Introduction

Axon elimination with or without the death of the cell body is a key feature in many neurological disorders and in response to nerve injury and chemical insults. In addition, neurites are eliminated by pruning during development, a key process in the wiring of the nervous system^1,2^. Both during development and in response to insults, axons are eliminated through a discrete series of events that ends with the clearance of the remnants by glia or other engulfing cells^3^. Clearance of the debris is crucial for the recovery of the degenerated axon. When clearance does not occur properly, it can lead to tissue scarring and inflammation, preventing regeneration and causing developmental defects^3,4^.

Phosphatidylserine (PS) is a phospholipid distributed exclusively on the inner leaflet of the cell membrane. Upon induction of cell death, PS is flipped to the outer membrane and acts as an “eat me” signal to recruit phagocytes^5–7^. In mammals, PS is recognized either directly or through a bridging molecule by several engulfment receptors expressed on phagocytes, triggering engulfment of the cell debris^8–16^. Multiple membrane proteins tightly control the distribution of PS under normal conditions and upon cell death. Flippases keep PS in the inner leaflet in an ATP-dependent manner and are inactivated during apoptosis. Scramblases, on the other hand, actively externalize PS from the inner leaflet to the outer leaflet^17–20^. PS receptors in *Drosophila melanogastar*, Zebrafish-*Danio rerio*, and mammals were shown to take part in axonal debris engulfment during pruning and in response to injury^10,21–26^, suggesting that PS serves as an “eat me” signal on degenerating axons. However, the pathways that control PS exposure have not yet been identified.

Using several in vitro paradigms of axonal degeneration, we show that PS is exposed on sub-axonal segments undergoing degeneration and that direct masking of the PS signal reduces axonal debris engulfment. Genetic and pharmacological manipulations of degeneration processes revealed that different pathways initiate PS exposure in different degeneration models. Interestingly, we found that Ca^2+^ influx is required for the execution of degeneration, but not for PS exposure, suggesting that there is divergence in pathways that control degeneration and PS exposure. Moreover, direct inhibition of axonal mitochondrial activity induced PS exposure without degeneration and elevation of the axonal energetic status-attenuated PS exposure. Overall, our results suggest that PS exposure can be regulated spatially on degenerating axons to mediate engulfment and can be decoupled from the degeneration process.

## Materials and methods

### Antibodies and reagents

Antibodies and dilutions used for immunofluorescence staining: mouse anti-Flag tag, M2 clone (Sigma-Aldrich, F1804, 1:500), mouse anti-SynCAM4/Necl4 (NeuroMab, UC Davis, 75–247, 1:500). Anti-mouse secondary antibody conjugated with Alexa 488 fluorophores were used at 1:500 (Jackson ImmunoResearch Laboratories). Phalloidin-TRITC were used at 1 μg/ml (Sigma-Aldrich, P1951).

Reagents: 20 mM NAD^+^ (nicotinamide adenine dinucleotide hydrate) (Sigma-Aldrich; N3014), 2 mM EGTA (Sigma-Aldrich, E4378), 5 μg/ml Z-VAD-FMK (Tocris, 2163), 10 mg/ml 10 kDa miniRuby-Dextran (Life Technologies, LSD3312), 5 μM Oligomycin A (Sigma-Aldrich, 55351), 10 μM FK866 (Sigma-Aldrich, F8557), 10 mM 2DG (2-Deoxy-D-Glucose) (Sigma-Aldrich, D8375).

### Mouse strains

Bax knockout (KO) mouse strain B6.129 × 1-Baxtm1Sjk/J (stock number 002994) was purchased from The Jackson Laboratory. Sarm1 KO mouse strain B6.129 × 1-Sarm1tm1Aidi/J (Stock Number 018069) was purchased from The Jackson Laboratory. Mice expressing the tdTomato fluorescent protein in their sensory neurons were generated by crossing Ai9 females (The Jackson Laboratory, stock #: 007909) with Brn3a^CRE-ER^ males. Timed pregnant females were subcutaneously injected with 110 μl of 20 mg/ml Tamoxifen (Sigma-Aldrich, T5648), dissolved in Corn oil (Sigma-Aldrich, C8267) at E12.5, 24 h prior to dissections.

### Production, purification, concentration, and quantification of recombinant ^FLAG^MFG-E8^D89E^

A plasmid containing the mutated form of MFG-E8 was gratefully received from Shigekazu Nagata, Kyoto Japan. Human embryonic kidney (HEK293T) cells were transfected with this plasmid using standard protocol and 24 h after transfection were cultured with serum‐free opti-MEM media. Conditioned media (CM) was collected 48 h later, and were 30 times concentrated using Vivaspin^®^ 20 tubes (Sartorius, 30,000 MWCO). The concentrated CM was dialyzed in dialysis tube (Tivan-Biotech, 3.5 kDa MWCO) overnight, in 2 l of PBS. For PS masking assay, CM was eluted on ANTI-FLAG M2 affinity gel beads (A2220, Sigma-Aldrich), with Flag peptide (F3290, Sigma-Aldrich).

### Explant culture

Dorsal root ganglion (DRG) explants of E13.5 mice were aseptically removed and cultured on poly-d-lysine (PDL)-laminin-coated plates. The explants were grown in Neurobasal^TM^-A (NB, GIBCO, 10888022) medium supplemented with 2% B-27, 1% glutamine, 1% penicillin–streptomycin, and 25 ng/ml mNGF 2.5 S (Alomone Labs; N-100) for 48 h to 120 h before treatments, one explant per well. For NGF deprivation, the medium was exchanged for medium lacking NGF with addition of 0.1 mg/ml mouse anti-NGF neutralizing antibodies (Alomone Labs; AN-240). For axotomy, axons were cut using a needle, in close proximity to the cell body. For vincristine treatment, the medium was exchanged to NGF containing medium, supplemented with 40 nM vincristine (LKT Labratories, V5254). For PS exposure assay, ^Flag^MFG-EE8^D89E^ was added to the medium, together with the treatment, at 1:200 concentration from concentrated ^Flag^MFG-EE8^D89E^ containing CM.

### Microfluidic cultures

DRGs from E13.5 mice were aseptically removed and pelleted in Hank’s balanced salt solution (Biological Industries) for 10 min and dissociated with 0.25% trypsin at 37 °C for 5 min. The trypsin was neutralized with 10 ml of L15 medium supplemented with 5% fetal bovine serum. The cells were then centrifuged at 2400 rpm at 21 °C for 4 min and resuspended in NB medium supplemented with B-27, glutamine, penicillin–streptomycin and 12.5 ng/ml NGF. The dissociated neurons were cultured on the PDL/laminin-coated microfluidic chambers and grown for 5 days as described^27^. In the axonal compartment, 50 ng/ml NGF were used. Axon degeneration was initiated in the axonal compartment after 5 days. For NGF deprivation, the medium was exchanged for medium lacking NGF with addition of 1 mg/ml of mouse anti-NGF neutralizing antibodies (Alomone Labs; AN-240) and ^Flag^MFG-EE8^D89E^. For vincristine treatment, the medium was exchange to NGF containing medium, supplemented with 40 nM vincristine (LKT Laboratories, V5254) and ^Flag^MFG-EE8^D89E^.

For PS masking assay, ^Flag^MFG-EE8^D89E^ at 10 μg/ml was added to axonal compartment.

### Quantification

#### PS exposure levels

In vitro cultures of DRG explants or MFC were co-stained with anti-Flag, anti-mouse 488 secondary antibodies, and phalloidin. Minimum of five separate explants were imaged and quantified per experimental condition. From each explant, 10 nonoverlapping frames were randomly collected. For each MFC experiment, three chambers were imaged and quantified per experimental condition, and eight nonoverlaping frames were randomly collected from each chamber. To evaluate PS exposure on the axons, the fluorescent intensities of each image were measured using ImageJ software (National Institute of Health). Fluorescent intensities were normalized to the area they cover.

#### Percentage of engulfing cells

In virto cultures of MFC (for NGF deprivation) or DRG explants (for axotomy) of tdTomato expressing DRG neurons and DRG glia were co-stained with mouse anti-Necl4 (glia cells marker) and Dapi (glia nuclei). tdTomato-positive neuronal debris that are engulfed are localized around the Necl4-positive glia cell nucleus. Percentage of engulfing cells were counted as the number of Necl4-positive cells that contained tdTomato debris around their nucleus from the total of Necl4-positive cells in each frame taken. For each experiment, 8–10 nonoverlaping frames were taken from each explant or MFC, and four explants and two MFC were imaged per experimental condition.

#### ATP levels

DRG explants were cultured on cell culture inserts system adequate to a six-well plate with 1-μm pore size coated on both sides with PDL and Laminin, allowing the axons to grow through the membrane (DRGs from 1.5 embryo per insert). After 48 h in vitro, both compartments were treated for indicated times. After treatment, axonal compartment material was collected and ATP levels were measured using ATP Bioluminescence Assay kit CLS II (ROCHE 11699695001) and ATP levels were normalized to protein levels in each sample.

#### Axon degeneration index

In vitro images of DRG explants, expressing tdTomato fluorescent protein, were binarized such that pixels corresponding to axons converted to white while all other regions converted to black. To perform this binarization and differentiate between axons and background in the images, a localized Otsu threshold was used. The Otsu algorithm searches for a threshold that minimizes the variance sum of two or more populations in an image^28^. This gives an exact threshold below which all pixels are considered background. This threshold was then applied to count the number of pixels corresponding to axons in each figure, which serves as the MTs stability index. A punctuated formation of MTs was evident from the DRG explants’ staining; these spots occupy only the higher gray levels in the image and appeared mostly in the NGF-deprived and not in their corresponding controls. The MT depolymerization index was defined as the ratio of depolymerized axon pixel number to intact axon pixel number. To detect the depolymerized axons, we used an algorithm for counting all the pixels above a certain threshold. To find this threshold, we calculated the probability density function (PDF) of the sum-controlled experiments (+NGF), from which the cumulative probability density function (CDF) was extracted. The threshold was set as the value above which there were almost no pixels (less than 0.1%). In each experiment, four explants in separate wells were imaged and quantified per experimental condition. From each explant, 10 nonoverlapping frames were randomly collected and imaged for quantification.

## Results

### PS is exposed on the membrane of degenerating axons

To systemically test the pathways that control PS exposure, we first screened for axonal exposure of PS in three in vitro models of axonal degeneration. Embryonic day 13.5 (E13.5) Dorsal Root Ganglia (DRG) explants were cultured for 48 h to 5 days before axon degeneration was initiated by either NGF deprivation, which mimcs axonal pruning^3,29,30^ or one of two insults: vincristine treatment or axotomy, that induces Wallerian degeneration^31,32^.

To mark PS exposure on axonal membrane, we used a mutated form of MFG-E8, a well-studied bridging molecule, that specifically binds PS and can not function as bridgining molecule^11,33^. The FLAG-tagged mutant MFG-E8 (^flag^MFG-E8^D89E^) was produced as previously described^11^ (see “Experimental procedures” section), and was added to the media concurrently with treatment inducing axonal degeneration. At the indicated time points, cultures were briefly fixed and exposed PS was identified by anti-FLAG staining without permeabilization. As expected, PS was not detected on the outer membrane of healthy axons. However, in all three degeneration paradigms, PS was detected on the outer membrane of the degenerating axons, distributed along the axon membrane, from soma to axon tip, although it appeared with different kinetics depending on treatment paradigm (Figure S1A–E). Increases in PS exposure became significant after 16 h of NGF deprivation or vincristine treatment, with a 10-fold increase in PS exposure measured after 24 h. In contrast, following axotomy, PS appeared on the distal axons as early as 2 h after the cut, with a 10-fold increase in PS by 16 h post-axotomy (Figure S1F).

To verify that the PS signal we measured reflects appearance of PS on the outer leaflet of the cell membrane and is not an artifact of penetration of the ^flag^MFG-E8^D89E^ into degenerating axons, we examined the ability of 10 kDa miniRuby-Dextran, a chemical agent that indicates membrane permeability, to penetrate axons in our in vitro models. We did not detect dextran staining within the axons for any of our treatments, supporting the conclusion that ^flag^MFG-E8^D89E^ staining in our studies identified solely extracellular PS (Figure S1G).

These results demonstrate that PS is exposed on the external membrane of degenerating axons for all three in vitro models of axonal degeneration, suggesting it may be important for recognition of degenerating axons in both Wallerian degeneration and pruning.

### PS is exposed on sub-axonal segments

Previous work showed that PS exposure can be restricted to specific segments of adult  cortical neurons undergoing apoptosis initiated by amyloid-β (Aβ) treatment^34^, but, whether PS can also be exposed on sub-axonal segments undergoing pruning is not known.

**Fig. 1:**
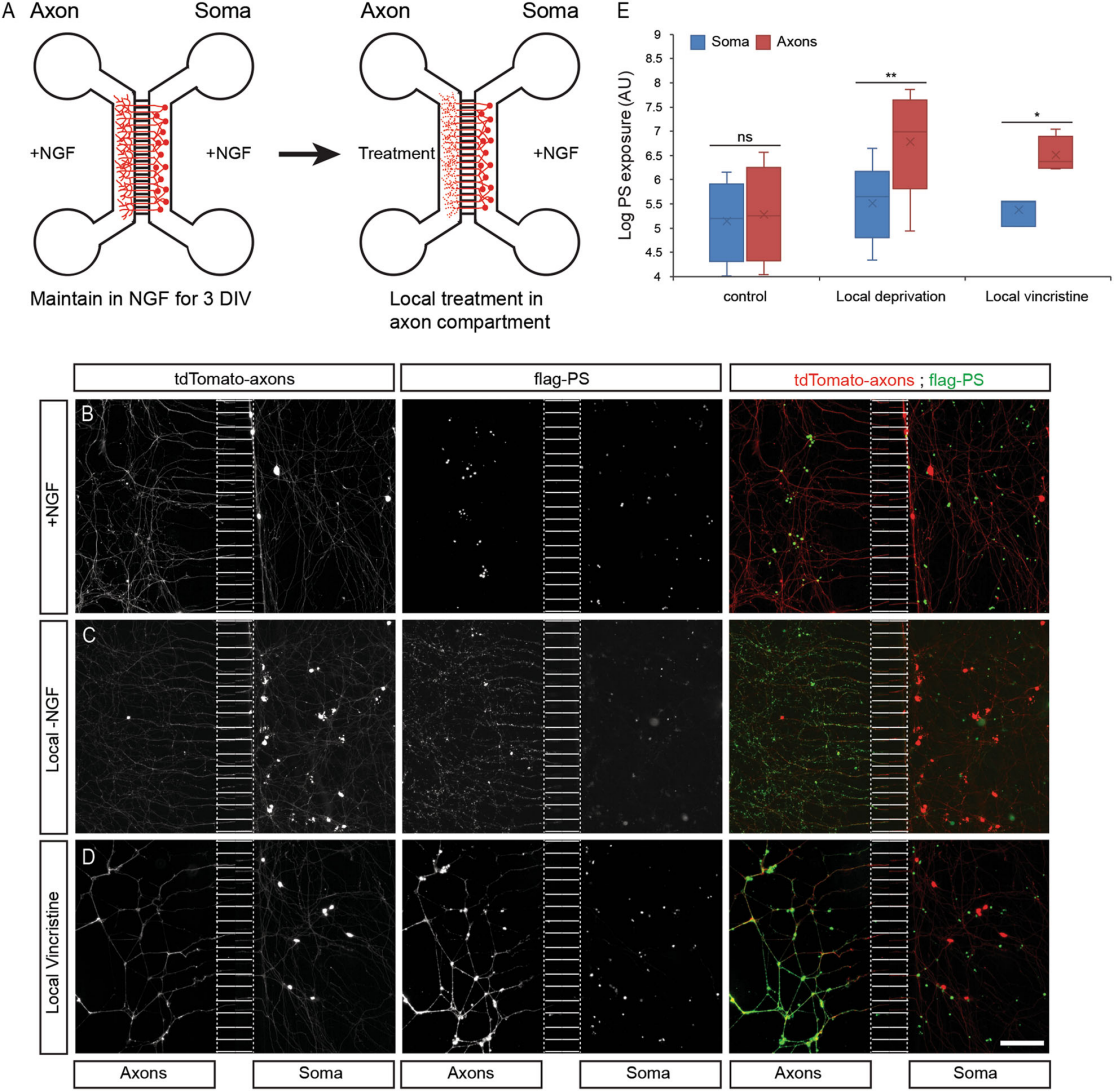
PS is exposed on sub-axonal segments. **a** Schematic representation of microfluidic chambers: axons and cell bodies are in separate compartments, allowing selective treatment of the axonal compartment. Dissociated tdTomato-positive DRG neurons were cultured in microfluidic chambers. After 5 days in vitro (DIV), the axonal compartment was treated, as indicated, for 24 h, with addition of ^flag^MFG-E8^D89E^. After treatment, cells were briefly fixed, stained with anti-Flag, and PS exposure was measured by anti-Flag staining intensity. **b** In control untreated cultures, the axons and cell bodies remained intact, and PS was not detected on the outer membrane. **c**, **d** Local axonal degeneration induced by NGF deprivation (**c**) or 40 nM vincristine treatment (**d**) for 24 h resulted in PS exposure on the treated distal axonal segment but not on the soma/proximal axons. **e** Quantification of PS exposure levels on the soma and axonal compartment in control and local axon degeneration. Error bars show mean ± SEM, *p*-value (student *t* test): *P < 0.05, **p < 0.01. Scale bar: 50 μm, *N* = 3 chambers per treatment

For these studies, we genetically marked DRG sensory neurons with tdTomato (see “Experimental procedures” for details). Dissociated tdTomato-positive DRG neurons from E13.5 embryos were cultured in microfluidic chambers, separating soma and axons to assess them in local axonal degeneration paradigm (Fig. 1a). Upon induction of local axon degeneration by NGF deprivation in the axonal compartment, PS exposure on distal axons increased by 4-fold (Fig. 1c–e). The chemotherapeutic agent vincristine caused PS exposure on distal axons as well, with 5-fold increase, compared to the control (Fig. 1d, e). In contrast, no PS exposure was measured in the soma/proximal axon compartment (Fig. 1b, e), indicating that PS exposure can be targeted to selective parts of the  cell membrane. These results reveal a potential role for PS exposure during the assembly of neuronal circuits, where it might control the removal of short neurites without damaging the surviving neuron.

### Blocking PS accessibility inhibits engulfment of axon remnants in vitro

**Fig. 2:**
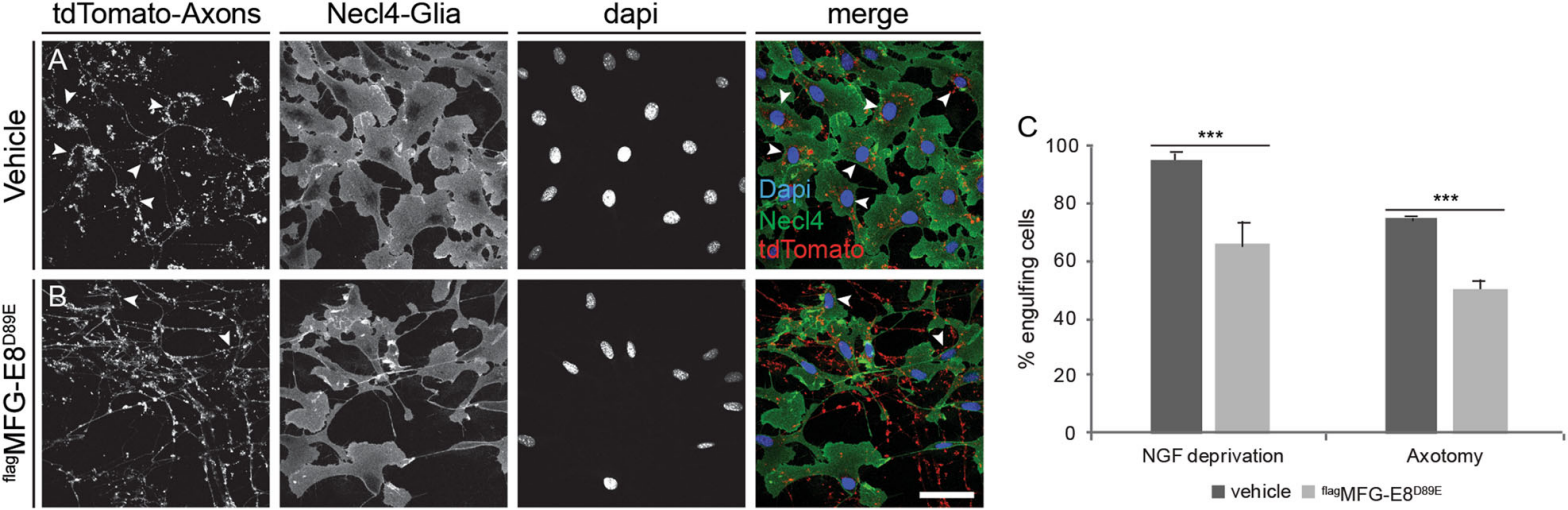
Masking PS signal reduces axonal debris engulfment. **a**, **b** tdTomato-positive DRG neurons were cultured in MFC for 5 days or as explants for 48 h, before being NGF-deprived for 24 h or axotomized for 16 h (Figure show NGF-deprived axons), with (**b**) or without (**a**) 10 μg/ml purified ^flag^MFG-E8^D89E^. White arrowheads marks Necl4/Td tomato double positive cells. **c** Quantification of percentage of engulfing glia cells. To evaluate engulfment of axonal debris, we counted labeled cells to determine the percentage of Necl4-positive/TdTomato debris-positive glia cells as a fraction of all Necl4-positive cells. Error bars mean ± SEM, *p*-value (student *t* test): ***p < 0.001. Scale bar: 50 μm, *N* = 3 chambers for NGF deprivation, four separate explants for axotomy

Our DRG neuronal cultures contain glia-like cells (Schwann cell precursors) identified by the Schwann cell marker Necl4 (Fig. 2). Interestingly, upon axonal degeneration, we detected tdTomato-positive debris within Necl4-positive cells, surrounding the cell nucleus, suggesting that these cells serve as local phagocytes (Fig. 2a). This allowed us to test the function of PS in axonal engulfment.

To study whether PS serves as an “eat me” signal on degenerating axons, we used the ^flag^MFG-E8^D89E^ to mask the PS signal as was previously done with apoptotic cells^33^ and evaluate debris engulfment by glial cells. For these studies, we immunopurified the ^flag^MFG-E8^D89E^ from CM as described^33^, and applied it to microfluidics cultures when axon degeneration was initiated by NGF deprivation (Fig. 2b, c), or to axotomized explant cultures (Fig. 2c). Engulfment was measured by the co-localization of tdTomato-positive axonal debris within Necl4-positive cells. When degeneration was induced by local NGF deprivation or axotomy in the absence of ^flag^MFG-E8^D89E^, about 95 and 70% of the glial cells were positive for axonal debris, respectively (Fig. 2c). Addition of purified ^flag^MFG-E8^D89E^ reduced this by ~30% (Fig. 2c), suggesting that engulfment requires PS exposure.

### PS exposure is controlled by early activators of axonal degeneration pathways

**Fig. 3:**
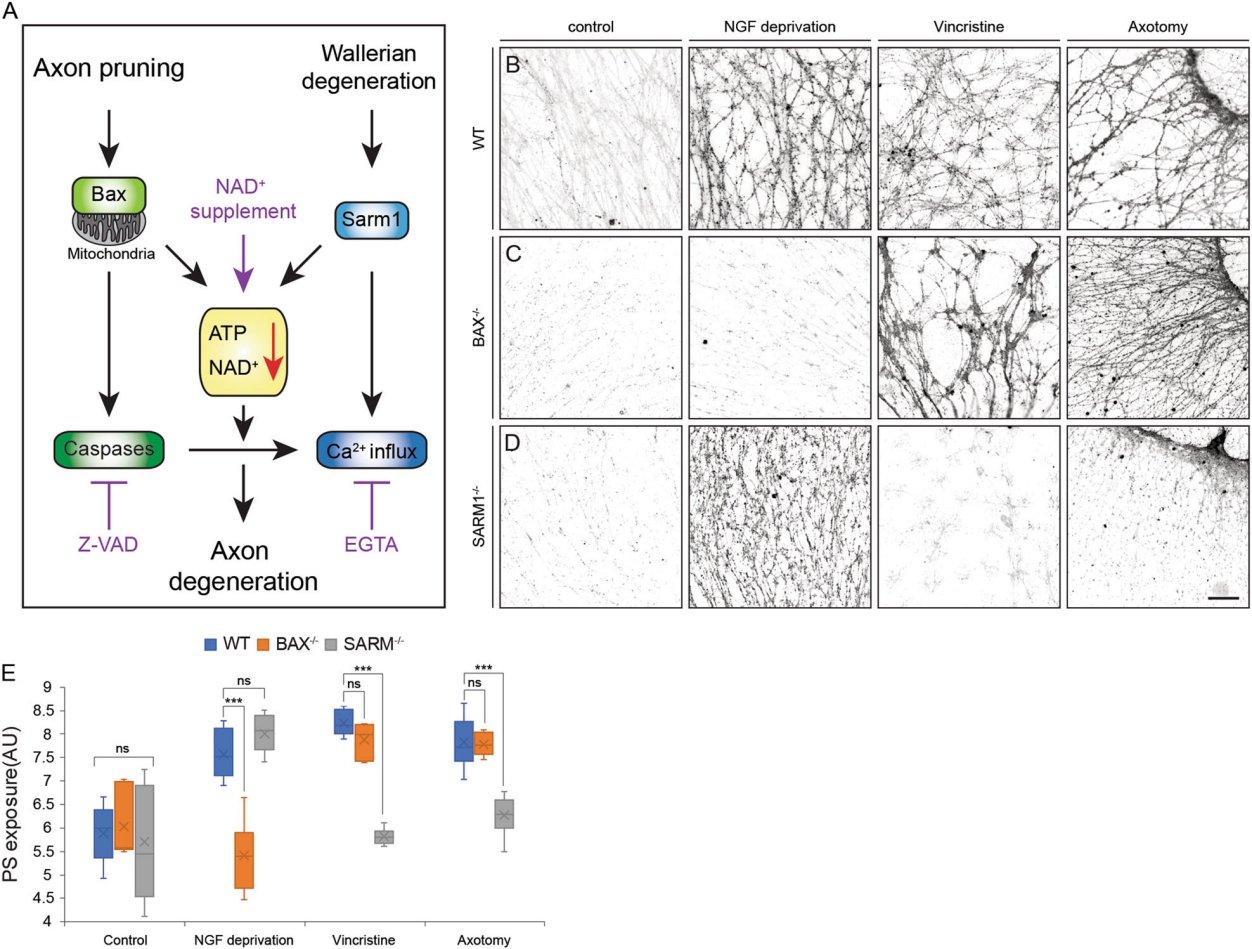
Early activators of axonal degeneration control PS exposure. **a** Schematic representation of the pathways that control axonal degeneration. Key activators of each pathway, as well as other downstream contributors to the pathways are depicted. Pharmacological treatments used in the experiments are marked in purple. **b-d** DRG explants of WT (**b**), Bax^-/-^ (**c**) and Sarm1^-/-^ (**d**) embryos were cultured for 48 to 96 h in the presence of NGF before axon degeneration was initiated by NGF deprivation, 40 nM vincristine, or axotomy, with addition of ^flag^MFG-E8^D89E^, for additional 16 h (Axotomy) or 24 h (NGF deprivation and Vincristine). After treatment, cells were briefly fixed, stained with anti-Flag, and PS exposure was measured by anti-Flag staining intensity. WT axons expose PS after all treatments, while Bax null axons expose PS after vincristine and axotomy, but not after NGF deprivation. Sarm1 null axons expose PS after NGF deprivation but not after vincristine or axotomy. **e** Quantification of PS exposure levels on WT, Bax^-/-^ and Sarm1^-/-^ axons in all treatments. Error bars mean ± SEM, *p*-value, compare with WT exposure levels (student *t* test): ***P < 0.001. Scale bar: 50 μm, *N* = minimum of five separate explants were analyzed per experimental condition

Since we detected PS exposure in all of our paradigms of axon degeneration, we aimed to identify the molecular mechanism that controls PS exposure in each one (Fig. 3a).

BAX is a pro-apoptotic protein that is activated early in trophic factor withdrawal-initiated degeneration and Bax null neurons are completely protected from NGF deprivation-induced axonal degeneration^35^. Similarly, Sarm1 is activated early during Wallerian degeneration and Sarm1 null mice are protected from vincristine-induced neuropathy and from distal axon degeneration after injury^36^.

To study the involvement of these two proteins in PS exposure during axonal degeneration, DRG explants from Bax^-/-^ and Sarm1^-/-^ embryos were NGF-deprived, vincristine-treated, or axotomized, and PS exposure was measured as described above. As expected, Bax^-/-^ neurons were protected from degeneration after NGF deprivation, and we found that the intact Bax^-/-^ neurons did not expose PS on their external membranes. In contrast, Bax^-/-^ neurons were not protected from vincristine or axotomy, and under these conditions, PS appeared on the external membrane to the same extent as in WT neurons (Fig. 3b, c, e). We also looked at PS exposure in Sarm1^-/-^ neurons. These neurons were protected from both axotomy- and vincristine-induced degeneration, and PS exposure was barely detected on the treated axons under these conditions. However, as previously shown, the Sarm1^-/-^ neurons were susceptible to NGF deprivation-induced degeneration. We found that NGF-deprived Sarm1^-/-^ neurons did expose PS on their outer membrane, similar to WT axons (Fig. 3b, d, e).

Overall, these results show that inhibiting axon degeneration by deletion of early activators in either pathway also blocks PS exposure to the outer leaflet of the axonal membrane.

### Blocking extracellular Ca^2+^ influx inhibits degeneration but does not prevent PS exposure

**Fig. 4:**
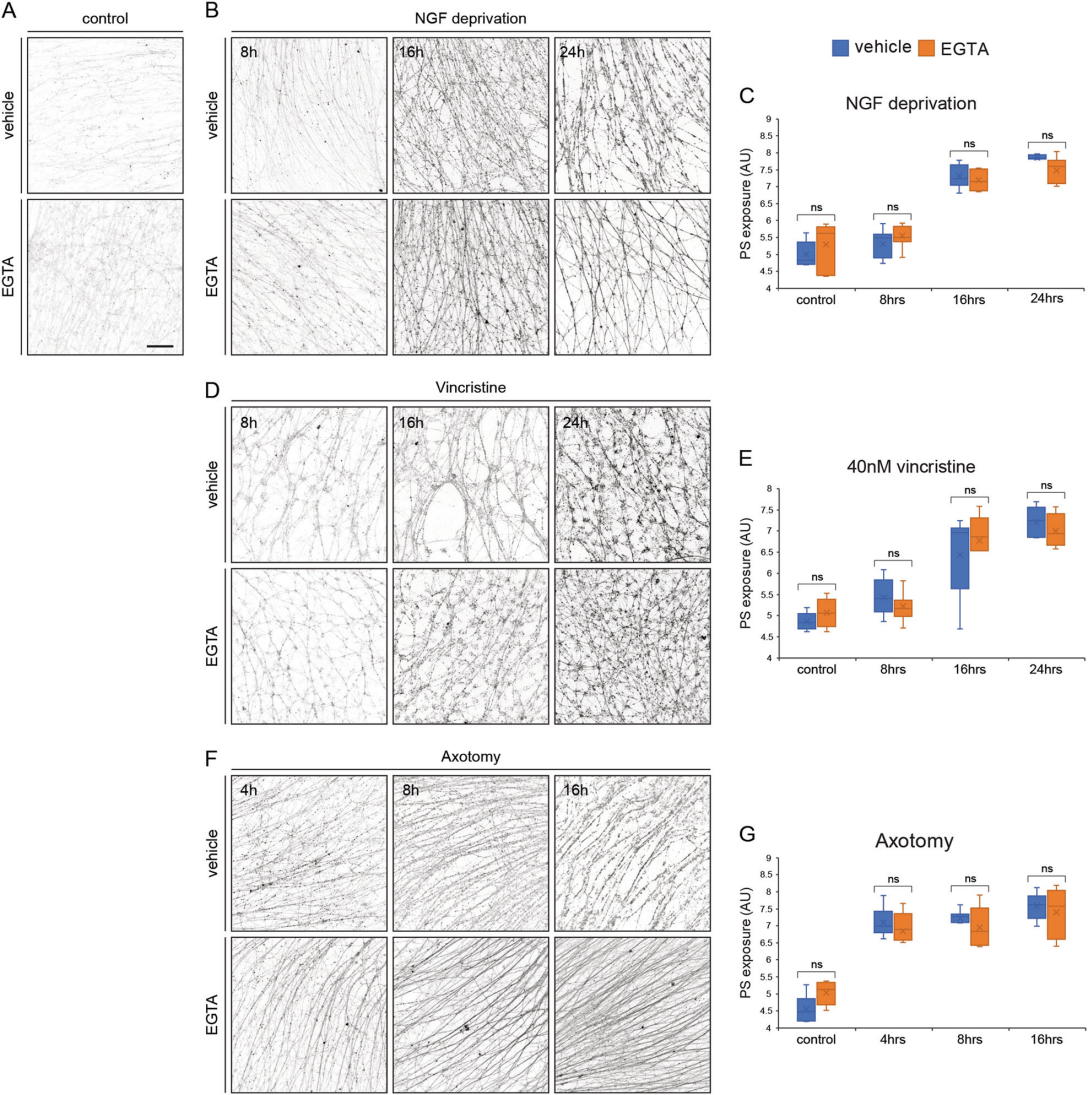
Blocking extracellular Ca^++^ influx does not prevent PS exposure. **a** DRG axons were cultured for 48 h in the presence of NGF before treatment with 2 mM EGTA for 24 h, with addition of ^flag^MFG-E8^D89E^. After treatment, cells were briefly fixed, stained with anti-Flag, and PS exposure was measured by anti-Flag staining intensity. EGTA had no effect on the basal levels of PS exposure. **b-g** DRG axons were cultured for 48 h and then treated as indicated in the presence of vehicle (upper rows) or 2 mM EGTA (lower rows). **b**, **c** EGTA had no effect on the exposure of PS in DRG axons deprived of NGF for 8, 16, or 24 h. **d**, **e** EGTA had no effect on the exposure of PS in DRG axons treated with vincristine for 8, 16, or 24 h. **f**, **g** EGTA had no effect on the exposure of PS in axotomized DRG axons at 4, 8, or 16 h post-axotomy; however, it completely protected axons from degeneration. **c**, **e**, **g** Error bars indicate mean ± SEM, significance determined by two-way ANOVA, Scale bar: 100 μm, *N* = minimum of five separate explants were analyzed per experimental condition

To further study the involvement of known axonal degeneration signaling pathways in PS exposure, we used chemical reagents to inhibit other downstream steps in the degeneration process. Extracellular calcium influx activates cell proteases in both NGF deprivation-induced degeneration and Wallerian degeneration, which takes part in cytoskeleton degradation during axon degeneration^31,37,38^. To block calcium influx, we treated the neurons with 2 mM EGTA, a calcium chelator (Fig. 3a). EGTA treatment was previously shown to protect axons from axotomy-induced degeneration, however, it was not clear whether it also protects against degeneration induced by other insults^37,39^. Thus, we first tested the effect of EGTA on axonal degeneration in all three degeneration models. As described before, EGTA treatment protected distal axons from degeneration after axotomy (Figure S2C, H). EGTA treatment did not protect NGF-deprived or vincristine-treated axons from degeneration, as measured by tdTomato-positive axons morphology (Figure S2C, F–G). However, we noted that EGTA partially protected the NGF-deprived axons, as their membrane remained mostly intact as visualized by the anti-Flag PS staining (Fig. 4b). Surprisingly, EGTA had no effect on PS exposure in any of these three in vitro degeneration models, thereby decoupling PS exposure from correlation with axon degeneration (Fig. 4). Although axotomized axons remained intact in the presence of EGTA even at 16 h post-axotomy, they still strongly exposed PS on the outer membrane (Fig. 4f, g). These results indicate that extracellular Ca^2+^ influx is not necessary for PS exposure and demonstrate that PS exposure can be separated from axonal degeneration.

### Caspases are involved in PS exposure only in apoptotic-dependent axonal degeneration

**Fig. 5:**
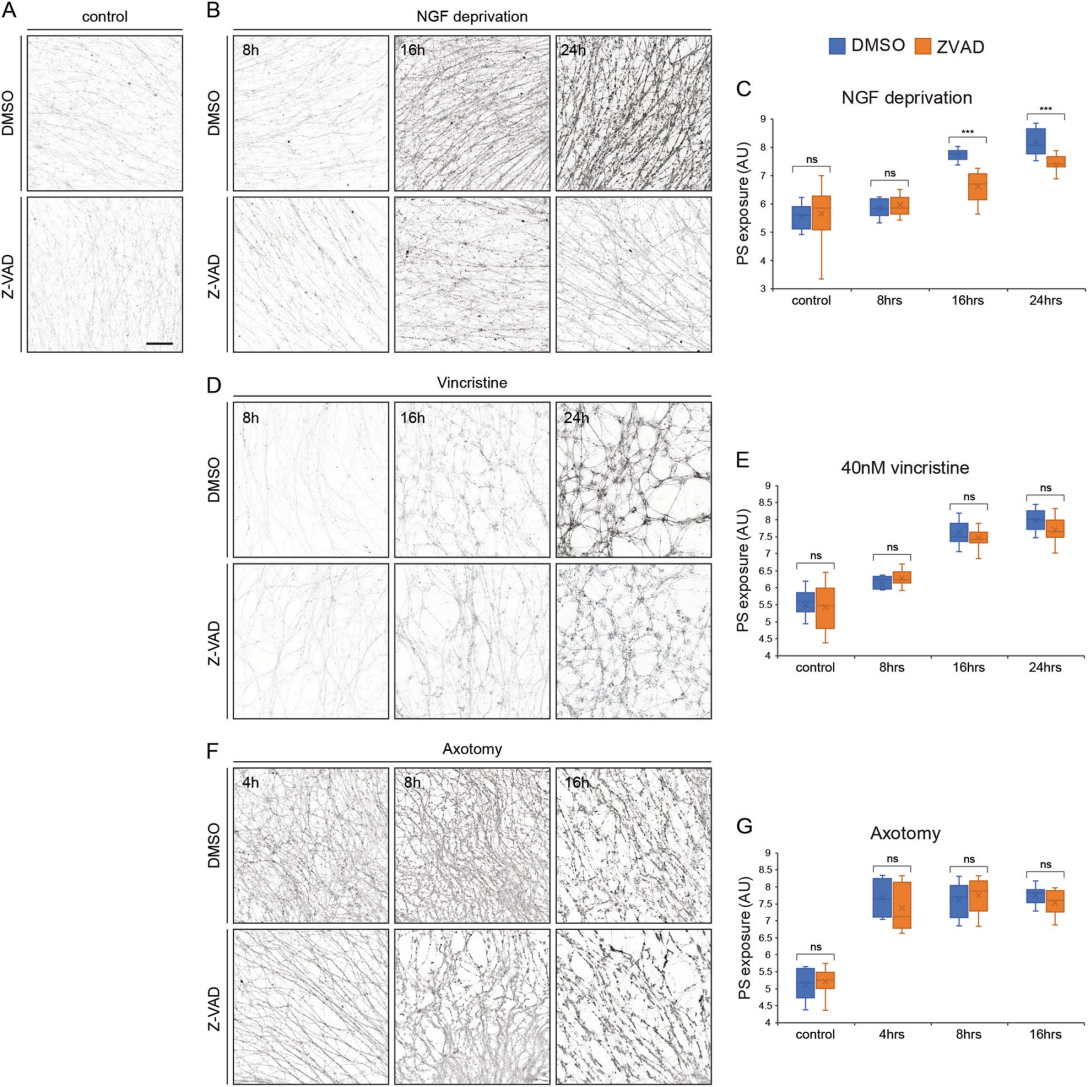
Inhibiting caspase activity prevents PS exposure only in apoptotic-dependent axon degeneration. **a** DRG axons were cultured for 48 h in the presence of NGF before a 24 h treatment with the pan-caspase inhibitor Z-VAD (50 μM) or vehicle (DMSO) with addition of ^flag^MFG-E8^D89E^. After treatment, cells were briefly fixed, stained with anti-Flag, and PS exposure was measured by anti-Flag staining intensity. Z-VAD had no effect on basal levels of PS exposure. **b**, **c** DRG axons were cultured for 48 h and then NGF-deprived for 8, 16, or 24 h with DMSO or 50 μM Z-VAD. PS exposure was significantly reduced after 16 h and 24 h of Z-VAD treatment. **d**, **e** DRG axons were cultured for 96 h and then treated with 40 nM vincristine with DMSO or Z-VAD for 8, 16, or 24 h. Z-VAD treatment did not prevent PS exposure on vincristine-treated axons at any time point. **f**, **g** DRG axons were cultured for 48 h before axons were axotomized using a sharp needle and cultured with DMSO or Z-VAD for 4, 8, or 16 h. Z-VAD treatment did not prevent PS exposure on axotomized axons at any time point. Error bars indicate mean ± SEM, *p*-value (two-way ANOVA): ***P < 0.001. Scale bar: 100 μm, *N* = minimum of five separate explants were analyzed per experimental condition

We next tested the role of caspases that act downstream of BAX in the apoptotic  pathway. Caspases are essential for axonal degeneration induced by trophic factor withdrawal^40^. To study the involvement of caspases in PS exposure during axon degeneration, neurons were treated with 50 μM Z-VAD-FMK (Z-VAD), a pan-caspase inhibitor (Fig. 3a). As shown previously, Z-VAD protects axons from degeneration after NGF deprivation but not after axotomy or vincristine treatment (Figure S2D, F, H). Treatment with Z-VAD reduced PS exposure on NGF-deprived axons by approximately 50% after 16 h and 24 h of deprivation, compared to DMSO-treated axons (Fig. 5b, c), while PS exposure levels on vincristine-treated and axotomized axons were not affected by Z-VAD treatment at any time point (Fig. 5d–g).

These results indicate that caspase activation during apoptosis-dependent axon degeneration is partially required for PS exposure.

### NAD^+^ supplementation inhibits PS exposure on degenerating axons

**Fig. 6:**
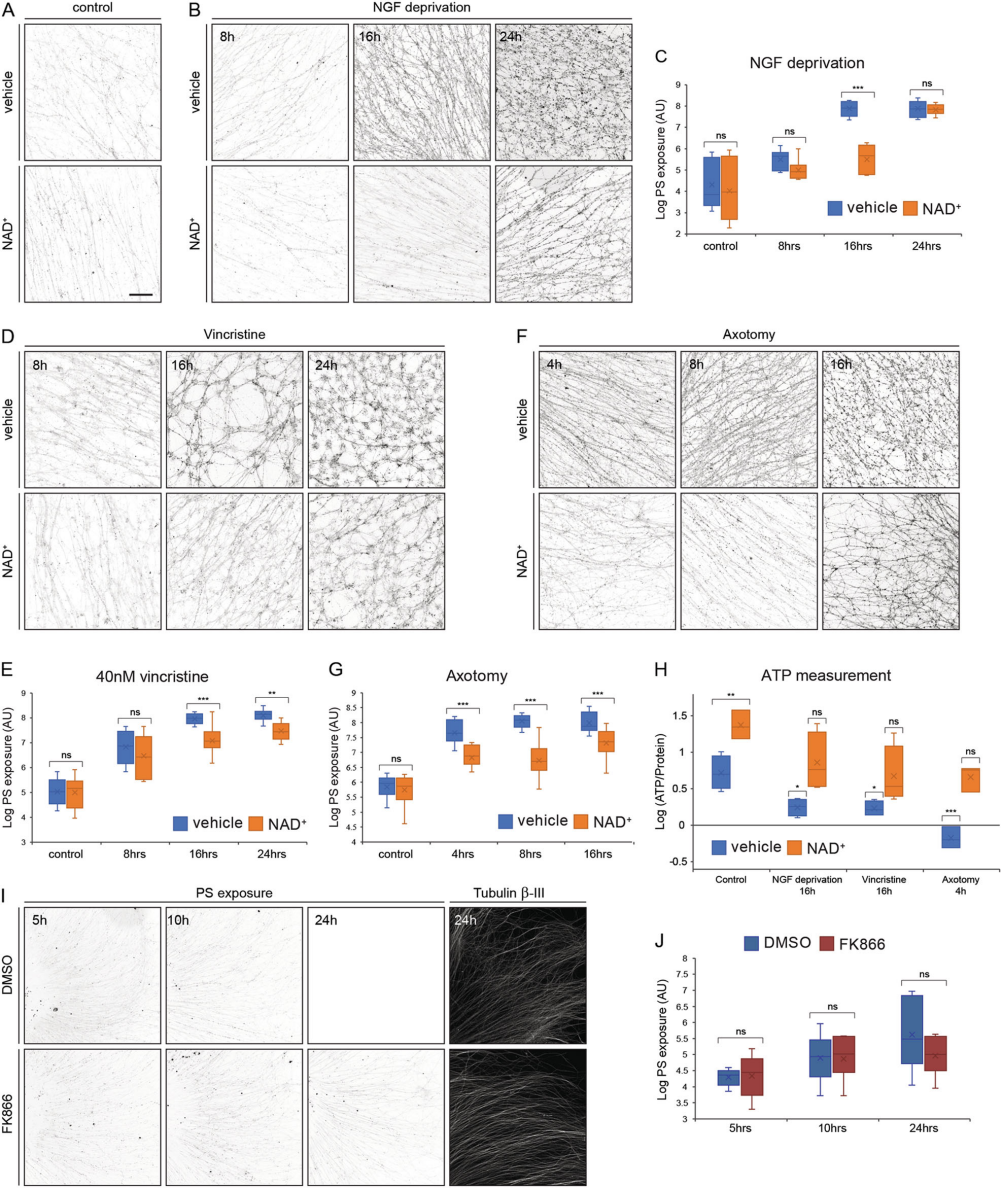
NAD^+^ supplementation suppresses PS exposure on degenerating axons. **a** DRG axons were cultured for 48 h in the presence of NGF before supplementation with 20 mM NAD^+^ or vehicle control and an additional 24 h of culture, with addition of ^flag^MFG-E8^D89E^. After treatment, cells were briefly fixed, stained with anti-Flag, and PS exposure was measured by anti-Flag staining intensity. NAD^+^ had no effect on basal PS exposure levels. **b**, **c** DRG axons were cultured for 48 h and then NGF-deprived for 8, 16, or 24 h with vehicle control or 20 mM NAD^+^ supplement. PS exposure was reduced significantly after 16 h in the NAD^+^ treated axons, but not at 24 h. **d**, **e** DRG axons were cultured for 96 h before treatment with 40 nM vincristine with vehicle or 120 mM NAD^+^ supplement for 8, 16, or 24 h. PS exposure was reduced significantly after 16 h and 24 h. **f**, **g** DRG axons were cultured for 48 h before axons were axotomized using sharp needle, and cultured with vehicle or 20 mM NAD^+^ supplement for 4, 8, or 16 h. NAD^+^ supplement prevented PS exposure on axotomized axons in all tested times. **h** ATP levels with or without NAD^+^ supplement. DRG explants were cultured on cell inserts for 48 h before treated with NGF deprivation, vincristine or axotomy. After indicated time points, axonal compartment were collected and ATP levels were quantified. All three treatments significantly reduced axonal ATP levels, compare with control. NAD^+^ supplement prevented ATP reduction and rescued axonal ATP levels back to control levels. **i** DRG axons were cultured for 48 h and then treated with 10 μM FK866 or DMSO for 5, 10 and 24 h. PS exposure was not affected by FK866 treatment at all time point tested. **j** Quntification of PS exspoure levels after FK866 or DMSO treatment. Error bars indicate mean ± SEM, *p*-value (two-way ANOVA): *P < 0.05, **P < 0.01, ***P < 0.001 (In H all *p*-values are compared to vehicle control). Scale bar: 100 μm, *N* = minimum of five separate explants were analyzed per experimental condition

Previous studies indicated that both apoptotic-dependent axon degeneration and Wallerian degeneration are NAD^+^-sensitive processes and supplementing axotomized axons or NGF-deprived axons with NAD^+^ protected them from degeneration^41–44^. To study whether NAD^+^ depletion contributes to PS exposure in degenerating axons, NGF-deprived, vincristine-treated, or axotomized axons were supplemented with 20 mM NAD^+^ (Fig. 3a). NAD^+^ protected the axons from NGF deprivation and axotomy, but not from vincristine-induced degeneration (Figure S2E, F, H). In contrast, NAD^+^ had similar effects on PS exposure for all three treatments. NAD^+^ supplement significantly reduced PS exposure on NGF-deprived axons after 16 h. However, this reduction was lost after 24 h of deprivation (Fig. 6b, c). This short-term effect may be due to depletion of NAD^+^ from the medium. The reduction in PS exposure on vincristine-treated axons was first observed after 16 h of treatment and was maintained at 24 h of treatment (Fig. 6d, e). Lastly, NAD^+^ supplement significantly reduced PS exposure on axotomized axons in all three time points tested (Fig. 6f, g). Overall, these results indicate that a drop in NAD^+^ during degeneration leads to PS exposure.

Energy levels in the axons were previously connected to the level of nutrients^39^. Therefore, we wanted to test whether NAD^+^ supplement rescues energy levels within the axons, which might also explain NAD^+^ protective abilities. ATP levels of NGF-deprived, vincristine-treated, and axotomized-DRG axons were measured with and without NAD^+^ supplement. NAD^+^ supplement rescued the drop in ATP levels in all conditions tested, as well as increasing ATP levels in control (Fig. 6h). These results show that NAD^+^ supplementation results in increase in ATP levels. Next, we tested whether direct reduction in intra-axonal NAD^+^ is sufficient by itself to induce PS exposure on the axons. FK866 is a pharmacological inhibitor of the enzyme NAMPT, an enzyme involved in biosynthesis of NAD^+^. Previous studies showed that 5 h treatment with FK866 reduced intra-axonal NAD^+^ levels by 60%, and 24 h of treatment reduced it by up to 90%^44–47^. Therefore, we treated DRG explant cultures for 5, 10, and 24 h with 10 μM FK866 or DMSO as control. FK866 treatment did not induce PS exposure on treated axons in any of the time-points tested. Furthermore, FK866 was not sufficient to induce axonal degneration, as was previously shown^46,48^ (Fig. 6i, j).

Taken together, our results show that intra-axonal reduction of NAD^+^ levels by itself is not sufficient to induce PS exsposure, suggesting, that PS exsposure is reagulated by the axonal energetic status.

### Inhibiting mitochondrial activity causes PS exposure

**Fig. 7:**
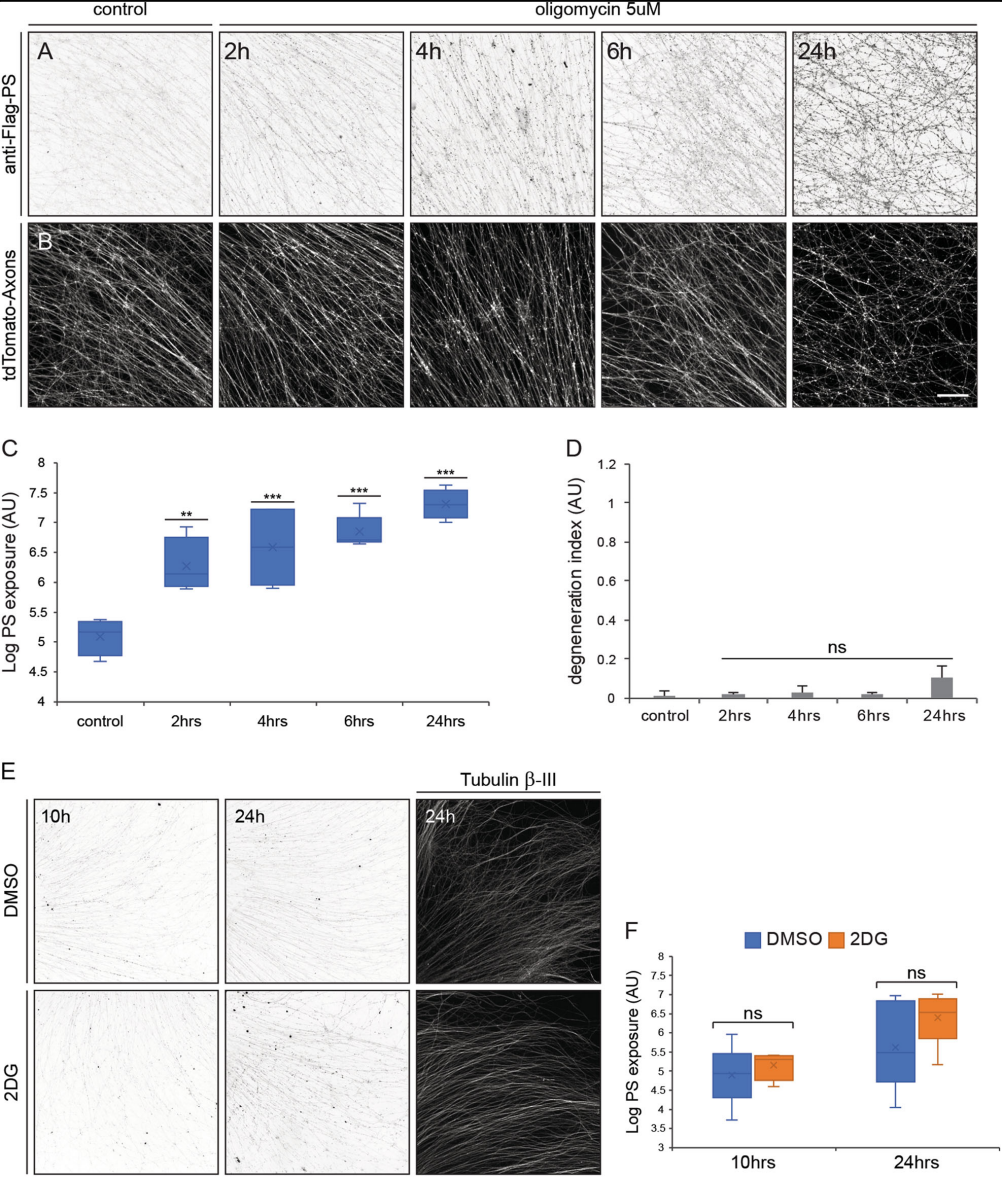
Inhibition of mitochondrial ATP synthesis leads to PS exposure without axon degeneration. DRG explants were cultured for 48 h before treatment with 5 μM Oligomycin for 2, 4, 6, or 24 h, with addition of ^flag^MFG-E8^D89E^. After treatment, cells were briefly fixed, stained with anti-Flag, and PS exposure was measured by anti-Flag staining intensity. PS exposure increased twofold after 2 h of treatment and increased to up to 10-fold at 24 h (**a**, **c**). Oligomycin-treated axons remained mostly intact, even after 24 h of treatment (**b**, **d**). **e** DRG explants were cultured for 48 h before treated with 10 mM 2DG or DMSO for 10 and  24h. 2DG treatment did not induce axonal degeneration. Twenty-four hours treatment resulted in a mild, yet not significant increase in PS exposure levels. **f** Quantification of PS exposure levels after 2DG and DMSO treatment. Error bars mean ± SEM, *p*-value (two-way ANOVA): **p < 0.01, ***P < 0.001. Scale bar: 100 μm, *N* = minimum of five separate explants were analyzed per experimental condition

 Since NAD^+^ supplies energy and metabolites, and due to the fact that PS maintenance in the inner membrane is an ATP-dependent process, we tested whether depletion of energy alone is sufficient to drive PS exposure. Mitochondria are the major source of axonal ATP, and inhibiting mitochondrial activity drastically reduces axonal ATP levels^39,49–51^. To study whether PS exposure can be induced by ATP depletion, DRG explants (soma and axons) were treated with Oligomycin, which inhibits mitochondrial ATP synthase. PS exposure was increased by 2-fold after 2 h of Oligomycin treatment, and the level of exposed PS increased by 10-fold after 24 h of treatment (Fig. 7a, c). Even though the PS exposure levels were similar to those measured after 24 h of NGF deprivation (Figure S1E), Oligomycin-treated axons degenerated much slower , and at 24 h, the majority of the axons were still intact (Fig. 7b, d). While mitochondria are the major source of axonal ATP, glycolysis  plays a role in axonal ATP production as well. 2-Deoxy-D-Glucose (2DG) is a direct inhibitor of glycolysis and previous studies showed that 2DG treatment reduced endogenous axonal ATP levels by about 30%, leading to changes in neuronal activity^52^. We treated DRG explant cultures with 10 mM 2DG or DMSO as control, for 10 and 24 h, and evaluated its effect on PS exposure. We did not detect an elevation in PS exposure above the vehicle control cultures (Fig. 7e, f). Moreover, in agreement with a previous report 2DG did not induce axonal degeneration^52^. Overall, these results further separate the mechanisms underlying axonal destruction and PS exposure and suggest that reduction in the mitochondrial ATP production can lead to PS exposure that precedes degeneration.

## Discussion

Axon elimination is a highly regulated process with critical roles in both health and disease. The removal of axonal debris is a key event, and is controlled both by the degenerating axon, which presents “eat me” signals, and by the engulfing cells that recognize these signals. While the molecular pathways that lead to destruction and fragmentation of the axons are being discovered, much less is known about the cross talk between the axon and the engulfing cell.

Our results demonstrate that PS exposure is a hallmark of axonal degeneration in all of the in vitro models we used. In addition, our demonstration of PS exposure occurring specifically in axonal sub-domains is a strong indication that there is a tight spatial regulation of PS exposure in the cell membrane, allowing it to serve as a selective “eat me” signal targeting axons or parts of axons for removal without cell death. This suggests that PS exposure could serve in other spatially-regulated processes executed by microglia and astrocytes, such as synapse elimination^53–56^.

Importantly, we show that masking PS with the dominant-negative form of MFG-E8 reduced the rate of engulfment of the axonal debris by Schwann cells, providing an evidence that PS serves as a bona fide “eat me” signal for degenerating axons in the PNS. Previously, activated microglia - the immune system of the brain, were shown to engulf PS-presenting CNS neurons^57,58^. Here, we show that the glia cells of the PNS can also act as local phagocytes and engulf degenerating neurons by identification of PS exposed on their membrane.

How is the exposure of PS regulated on the degenerating axons? Our genetic and pharmacological manipulations suggest that there is no single degenerative pathway that leads to the exposure of PS on the axons, and activation of both apoptosis-dependent and -independent degeneration results in PS exposure. Previous work showed reduced PS exposure on injured Wld^s^ retinal gangilion neurons, indicating the involvement of the Wallerian degeneration pathway in exposure of PS^59^. We were able to pinpoint the involvement of Sarm1 in PS exposure of injured and vincristine-treated DRG neurons, as well as NAD^+^, allowing better understanding of the mechanism involved in PS exposure during Wallerian degeneration. Interestingly, although manipulation of upstream elements, such as BAX and Sarm1, suggested a strong link between axonal breakdown and PS exposure, our manipulation of downstream events revealed a divergence in the pathways for PS exposure and axonal degeneration. The most striking effect is the inhibition of Ca^2+^ influx by EGTA, which preserved the axonal structure and membrane, but did not block exposure of PS on the outer membrane, supporting the idea that PS exposure is not a simple by-product of degeneration. Previous studies demonstrated that caspases regulate PS exposure through inactivation of the PS flippase, ATP11C, on one hand, and activation of XKRs scramblases on the other^19,20,60,61^. In agreement with this model, we detected a 50% reduction of axonal PS exposure following trophic withdrawal by the pan-caspase inhibitor Z-VAD. However, Z-VAD did not prevent PS exposure in the other models of axonal degeneration. This result suggests that caspase regulation of PS flipping enzymes is not a conserved mechanism for PS exposure in all the forms of axonal degeneration. A recent study implicated the involvement of specific scramblases in PS exposure on degenerated axons, such as Xkr8, in injury model of axonal degeneration^62^. However, to fully identify the enzymes that control PS exposure on degenerated axons in other models of degeneration, as well as injury, more in vivo and in vitro studies are necessary.

Another common feature in axonal degeneration is the depletion of NAD^+^ from the degenerating axons. Previous studies by others and us revealed that supplementing neurons with NAD^+^ either alone or in combination with Z-VAD protects against axonal degeneration^42,46,63,64^. Moreover, overexpression of the cytoplasmic Nmnat1 protects against trophic withdrawal-induced degeneration with no effect on caspase activity^44,65^. Here, supplementing with NAD^+^ significantly reduced PS exposure in all models of axon degeneration tested, suggesting that NAD^+^ plays a key role in PS exposure. The involvement of NAD^+^ depletion in PS exposure was also recently reported in *Drosophila Melongestar* injured or remodeling axons^66^. It is possible that depletion of NAD^+^ during axonal degeneration activates unknown downstream enzymes that actively expose PS to the outer leaflet, or that the depletion leads to reduction in the axonal energetic state, leading to changes in phospholipid distribution along the cell membrane due to reduced activity of the ATP-dependent flippase. In agreement with the latter, inhibition of mitochondrial activity was sufficient to expose PS on the outer membrane before axonal degeneration was detected. Therefore, it is highly possible that reduction in ATP as part of the degeneration processes inactivates the PS flippases, leading to changes in PS distribution and its accumulation on the outer membrane, without active enzymes to flip it back inside. In concord, reduction in endogenous NAD^+^ by itself, without initiation of axonal degeneration as was achieved by FK866 treatment, is not sufficient to induce PS exposure and axonal degeneration. Interestingly, FK866 was previously shown to protect axons from degeneration induced by injury, and this protection was attributed to FK866 inhibiting accumulation of nicotinamide mononucleotide (NMN, an NAD^+^ precursor)^44,47,67^. Therefore, the exact role of NAD^+^ in the regulation of PS exposure remains to be further studied.

It was recently reported that the autophagic machinery promotes both Wallerian degeneration and PS exposure^68^. Knocking down autophagy-related genes resulted in decrease in PS exposure on axotomized axons. However, the researchers reported that PS exposure was independent of mitochondrial ATP production, as PS exposure was maintained after treatment of Oligomycin. In our experimental settings, it seems that reduction of mitochondrial ATP production actually increases PS exposure even without axonal injury^68^. However, a previous study indicated that PS exposure after axotomy correlates with loss of mitochondrial membrane potential and not with elevation in ATP levels^69^. Moreover, it is established that ATP is required for PS internalization by PS flippases^6,7^. This is also supported by our results using Oligomycin. It is therefore likely that the reduction in PS exposure that was observed in autophagy-deficient axons is due to overall inhibition of the degeneration process.

Whether PS exposure by itself can trigger axon degeneration is not yet clear. One evidence for that being the case arises from the naturally occurring mutation in ATP8A2, a P-Type ATPase PS flippase, in the Wabbler-lethal (*wl*) mice^70^. This mutation results in elevated PS exposure and axonopathy - pronounced neurodegeneration in both the CNS and the PNS. While it is not clear whether the phenotype is a result of increased engulfment, it is tempting to speculate that the exposed PS makes the axon vulnerable to damage by engulfing cells^70–72^.

Altogether, our data provide a strong evidence that PS serves as an “eat me” signal on degenerating axons. Surprisingly, PS exposure can be induced without axonal degeneration by axonal mitochondrial inhibition. Since mitochondrial dysfunction is the basis of several neurodegenerative diseases^73^, PS exposure may contribute to disease progression by triggering pathological engulfment.

## Electronic supplementary material

Supp Figure 2

Supp Figure 1

supplementary figure legends
