# Supplementary figures and images for: Phosphatidylserine is a marker for axonal debris engulfment but its exposure can be decoupled from degeneration
Source: Cell Death Dis. 2018 Nov 2;9(11):1116. doi: 10.1038/s41419-018-1155-z (PMC6214901; doi:10.1038/s41419-018-1155-z)

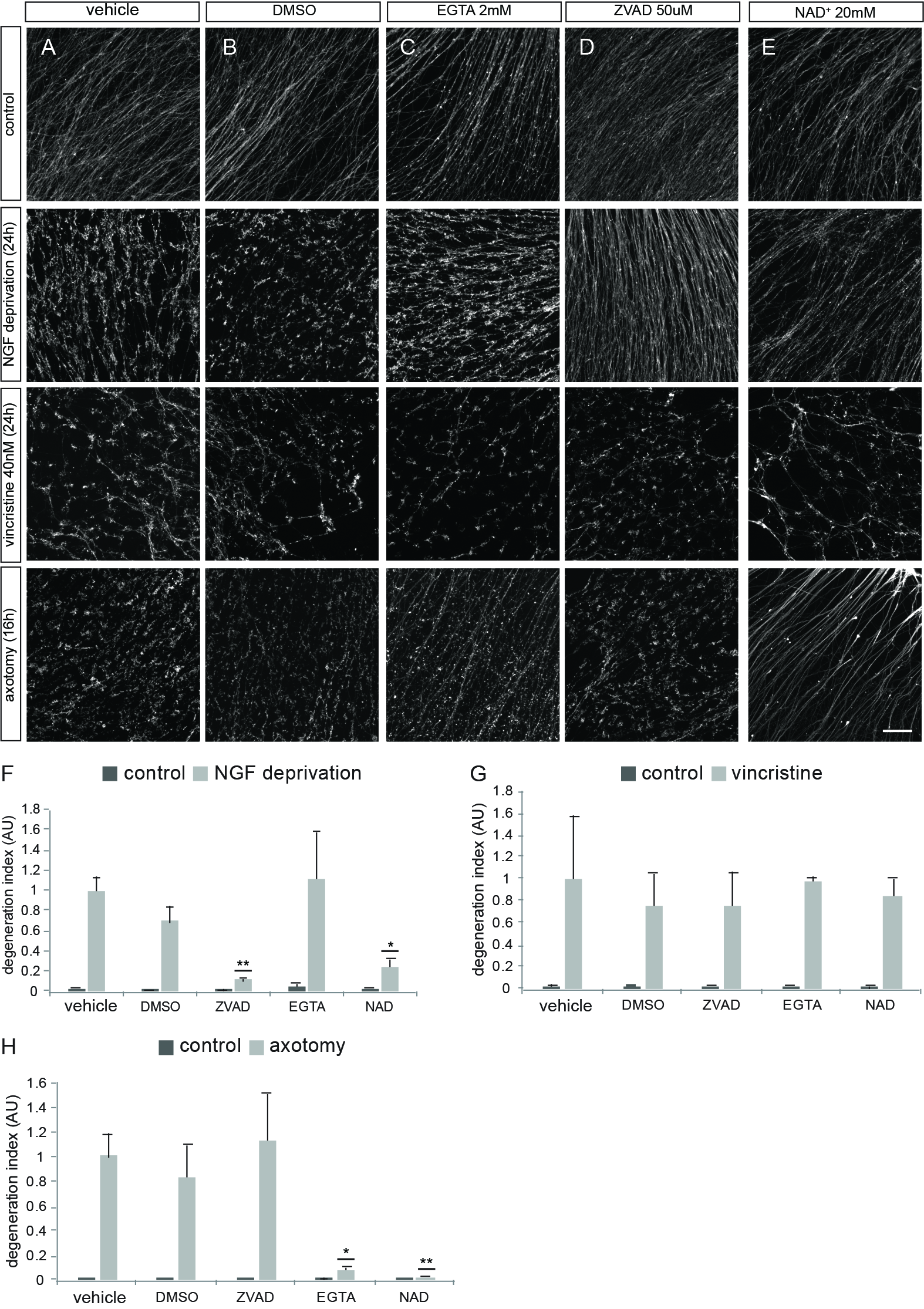

Supplement: Supplementary file 1 — Supp Figure 2 [file 41419_2018_1155_MOESM1_ESM.tif]

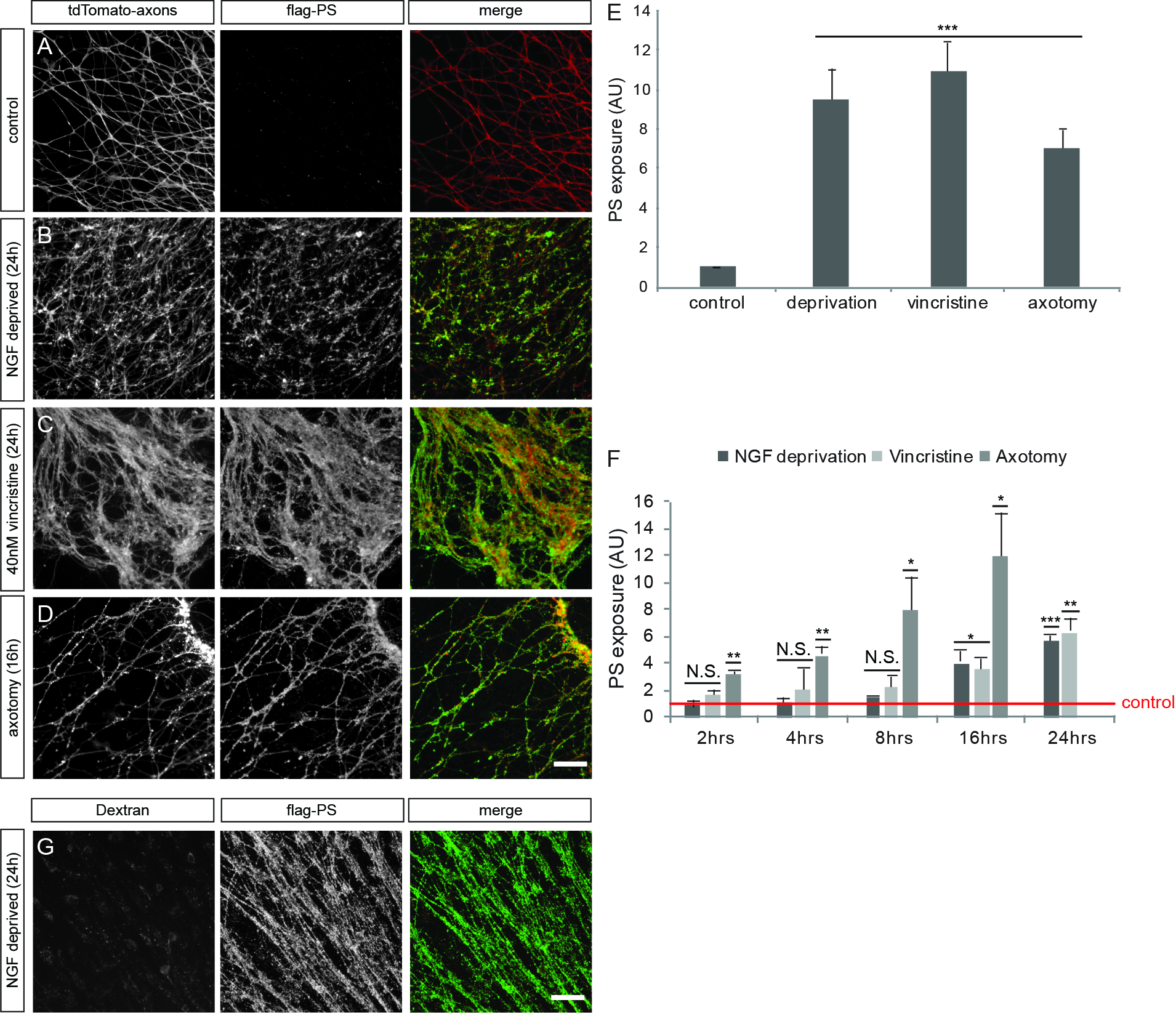

Supplement: Supplementary file 2 — Supp Figure 1 [file 41419_2018_1155_MOESM2_ESM.tif]
