# Supplementary material for: Phosphatidylserine is a marker for axonal debris engulfment but its exposure can be decoupled from degeneration
Source: Cell Death Dis. 2018 Nov 2;9(11):1116. doi: 10.1038/s41419-018-1155-z (PMC6214901; doi:10.1038/s41419-018-1155-z)
Supplement: Supplementary file 3 — supplementary figure legends [file 41419_2018_1155_MOESM3_ESM.docx]

**Figure S1. PS is exposed on degenerating DRG axons.** DRG explants were cultured in the presence of NGF before axon degeneration was initiated by NGF deprivation, 40nM vincristine treatment, or axotomy, with addition of ^flag^MFG-E8^D89E^. After treatment cells were briefly fixed, stained with anti-Flag and PS exposure was measured by intensity of anti-Flag staining. A. Intact axons exposed basal levels of PS and all exposure levels were normalized to PS levels on intact axons. B-E. Initiation of axon degeneration by 24h of NGF deprivation (B), 24h of vincristine treatment (C), and 16h after axotomy (D) resulted in approximately 10-fold increase in PS exposure levels compared to control (E). F. Time course analysis revealed different kinetics of PS exposure after the different treatments. Axotomy resulted in significant PS exposure by 2h after the cut, while NGF deprivation and vincristine treatments caused significant PS exposure increase only after 16h. G. Axons were treated with 10kDa fluorescent-dextran after NGF deprivation, vincristine treatment, or axotomy. The lack of fluorescence in the axons suggests that PS staining seen in B-F is not due infiltration of the ^flag^MFG-E8^D89E^ into the axons. Error bars - mean ± SEM, p-value (t-test)- *-P<0.05, **-P<0.01, ***-P<0.001, compared to PS levels at control (red line). Scale bar- 50μm, N= minimum of 5 separate explants were analyzed per experimental condition.

**Figure S2. Axon degeneration is controlled by two distinct pathways.** DRG explants were cultured under control conditions before axon degeneration was initiated by NGF deprivation, vincristine treatment or axotomy. The effect of DMSO (B), 2mM EGTA (C), 50μM Z-VAD (D) and 20mM NAD^+^ (E) ^on^ axon degeneration in each treatment was quantified. Z-VAD and NAD^+^ treatment protected NGF deprived axons from degeneration (D, E, F), while DMSO and EGTA did not affect axon degeneration (F). None of the treatments protected vincristine treated axons from degeneration (G). EGTA and NAD^+^ but not DMSO and Z-VAD, protected injured axons from degeneration (H). Results are represented as fold change in axonal degeneration compare to control cultures. Error bars - mean ± SEM, p-value (t-test)- *-P<0.05, **-P<0.01 (compare to results in vehicle control). Scale bar- 100μm, N= minimum of 5 separate explants were analyzed per experimental condition.
